# High-Dimensional Single-Cell Transcriptomics in Melanoma and Cancer Immunotherapy

**DOI:** 10.3390/genes12101629

**Published:** 2021-10-16

**Authors:** Camelia Quek, Xinyu Bai, Georgina V. Long, Richard A. Scolyer, James S. Wilmott

**Affiliations:** 1 Melanoma Institute Australia, The University of Sydney, Sydney, NSW 2006, Australia; xbai6546@uni.sydney.edu.au (X.B.); Georgina.Long@melanoma.org.au (G.V.L.); Richard.Scolyer@health.nsw.gov.au (R.A.S.); james.wilmott@sydney.edu.au (J.S.W.); 2 Faculty of Medicine and Health, The University of Sydney, Sydney, NSW 2006, Australia; 3 Charles Perkins Centre, The University of Sydney, Sydney, NSW 2006, Australia; 4 Royal North Shore and Mater Hospitals, Sydney, NSW 2065, Australia; 5 Tissue Pathology and Diagnostic Oncology, Royal Prince Alfred Hospital and NSW Health Pathology, Sydney, NSW 2050, Australia

**Keywords:** melanoma, single-cell, transcriptomics, sequencing, spatial, immunotherapy, cancer, treatment, diagnosis

## Abstract

Recent advances in single-cell transcriptomics have greatly improved knowledge of complex transcriptional programs, rapidly expanding our knowledge of cellular phenotypes and functions within the tumour microenvironment and immune system. Several new single-cell technologies have been developed over recent years that have enabled expanded understanding of the mechanistic cells and biological pathways targeted by immunotherapies such as immune checkpoint inhibitors, which are now routinely used in patient management with high-risk early-stage or advanced melanoma. These technologies have method-specific strengths, weaknesses and capabilities which need to be considered when utilising them to answer translational research questions. Here, we provide guidance for the implementation of single-cell transcriptomic analysis platforms by reviewing the currently available experimental and analysis workflows. We then highlight the use of these technologies to dissect the tumour microenvironment in the context of cancer patients treated with immunotherapy. The strategic use of single-cell analytics in clinical settings are discussed and potential future opportunities are explored with a focus on their use to rationalise the design of novel immunotherapeutic drug therapies that will ultimately lead to improved cancer patient outcomes.

## 1. Introduction

**Figure 1:**
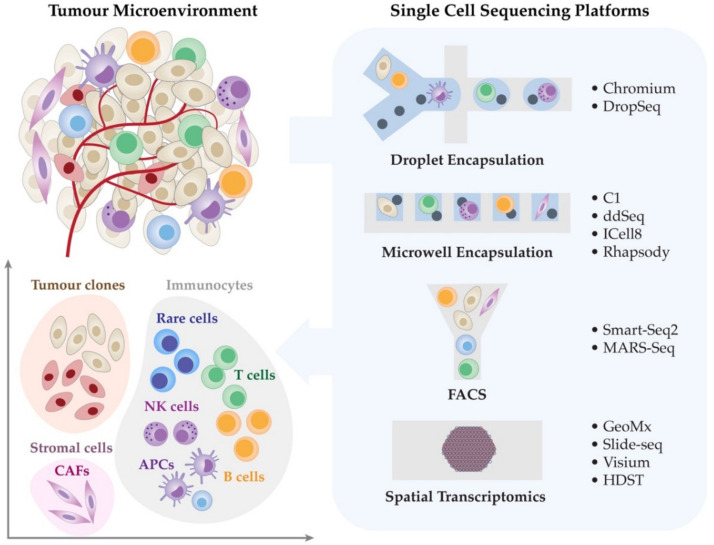
Single cell sequencing facilitates the dissection of the tumour microenvironment. The tumour microenvironment (TME), including malignant cells, immune cells and stromal cells, regulates a range of cellular and molecular signals. Such signals influence the cell states, tumour proliferation, host immunity and response to systemic therapies. Single cell technologies are providing unique opportunities for dissecting the orchestration of the TME and understanding the tumour-intrinsic and -extrinsic mechanisms of immunotherapy response and resistance.

Tumours are made up of a complex mixture of proliferating malignant cells, immune cells, blood vessels and tumour stroma (Figure 1) [1,2,3]. A network of pro-tumour and anti-tumour signals interact within the tumour microenvironment to modulate tumour growth and influence treatment response [4,5,6]. Immune checkpoint-based immunotherapies (ICIs) such as the antibodies that target immunomodulatory receptor, cytotoxic T-lymphocyte-associated protein 4 (CTLA-4), programmed cell death protein 1 (PD-1), or to its ligand, programmed death ligand 1 (PD-L1), have revolutionised the treatment of a variety of cancer types including melanoma [7,8,9,10,11]. The 5-year overall survival (OS) for Stage IV metastatic melanoma patients has improved from less than 10% to 50% with modern anti-PD-1-based immunotherapies when compared with historical data [7,8,12,13]. Despite these improvements, ~30–40% of patients do not respond and a further ~20–30% eventually relapse, and 50% die from their disease [12,13]. Although bulk genomic and transcriptomic data have provided valuable insights into the biological processes of treatment responses, the averaging of signals across millions of cells loses information about rare and unique cellular subtypes that might be pivotal in determining disease biology [14,15,16,17]. Single-cell analyses can provide unique opportunities to gain a deeper mechanistic understanding of tumour-intrinsic and -extrinsic mechanisms that drive response and resistance to immunotherapies [18,19,20].

Current approaches for single-cell analysis using immunohistochemistry, in-situ hybridisation, and flow cytometry have been essential tools for detecting the differences between non-malignant cells and cancer cells in the laboratory, as well as in the clinic [21,22,23,24]. These conventional methods examine individual cells and dissect the different subtypes of cells in the tumour and complement bulk-based genomic analysis [4,25]. However, such classic approaches often only detect a limited number of analytes in the assay, which reduces the power to characterise the diversity of cellular subtypes and molecular states in the TME [25,26,27].

Advances in single-cell transcriptomics technologies have empowered the unbiased detection of hundreds or thousands of analytes at single-cell resolution within many cells at an increasingly cost-effective manner. Likewise continual growth in the number of highly developed bioinformatics tools has empowered researchers with the opportunity to utilise single-cell technologies to fully characterise the diversity of different cell types and cell-to-cell interactions [28,29]. The analytical workflows include the reduction of high-dimensional data, neighbourhood clustering, phylogeny inference, lineage tracing, pseudotemporal ordering, RNA velocity, ligand–receptor interaction, and multiple data integration [28,29,30,31,32]. In addition, the sequencing-based mRNA molecules can be complemented by histological staining to further integrate cell locations and morphological features [33]. Recent single-cell-based studies of tumour cells and TME in melanoma and other cancer types have discovered new cellular subsets, unique transcriptional programs, and more evidence for “intra-tumoural” and “inter-tumoural” heterogeneity, all of which impact our understanding of therapeutic response and resistance [34,35,36,37]. For instance, advanced melanomas that have accumulated innate immunosuppressive cells such as myeloid-derived suppressor subpopulations respond poorly to immune checkpoint inhibitors [38,39,40]. Furthermore, varied infiltration of dysfunctional T cell subpopulations exhibits different levels of anti-tumour response [16,41,42]. Single-cell approaches offer great potential to answer many clinically and biologically important questions that persist in cancer research, including: the contribution of TME and tumour heterogeneity to evade the anti-tumour response; the temporal relationship between T cell clonotype and tumour cells during treatment; the interacting network of immune checkpoint molecules in TME; and the functional roles and spatial relationships between tumour cell subtypes and immune cells [43,44,45,46,47]. Herein, we focus on how single-cell technologies have the potential to advance understanding of the interplay between tumour cells and their microenvironment, and response and resistance to anti-cancer immunotherapy.

In this review, we summarise the landscape of commonly used single-cell and spatial transcriptomic technologies in cancer research, whilst discussing their advantages and shortcomings in terms of their capture efficiency, cell restriction, spatial resolution, and analytical support. Next, we describe the key discoveries and potential applications of single-cell techniques for novel biomarker and therapeutic development. Finally, we discuss the advances made using single-cell techniques in healthcare and clinical research.

## 2. Single-Cell Transcriptomic Technologies

In recent years, a wide array of single-cell transcriptomic technologies has emerged with a range of biological and clinical applications in melanoma and cancer immunotherapy, demonstrating the ability to characterise rare cellular phenotypes and specific cellular responses in an unbiased manner with precision [46,48,49,50]. Several recent studies have highlighted the impact of single-cell transcriptomics for identifying new potential molecular targets and the effect of checkpoint inhibitors on tumour cells and the TME [3,51,52,53]. These studies have fully characterised the cellular composition and function of the TME, T cell and immunosuppressive cell states, transcriptional programs and checkpoints associated with disease progression and response to treatment [3,17,51,52,53,54]. The single-cell transcriptomic sequencing technologies are broadly classified by their respective cell isolation methods (Table 1); (i) droplet encapsulation, (ii) microwell encapsulation, and (iii) fluorescence-activated cell sorting (FACS). In this section, we outline the following single-cell transcriptomic platforms including 10x Chromium, Fluidigm C1, and SMART-seq2, that have been widely used in the field of cancer research, thus providing a summarised guide that can assist a broad range of biomedical researchers to make an informed decision for their single-cell studies.

### 2.1. Droplet Encapsulation Technologies

The strength of droplet encapsulation technologies, 10x Genomics Chromium and Dolomite-Bio Nadia, is the ability to sort a large population of cells into a small volume of single cells within droplets, offering high-throughput and relatively low-cost analysis of single cells due to sample and reagent efficiency [55,56]. The Chromium system utilise GEM (Gel Bead-in-emulsion) technology in which a high diversity pool of gel beads, each coated with a unique oligonucleotide barcode sequence that are mixed with reverse-transcription (RT) reagents and cells in an oil environment to form thousands of individual cell emulsion droplets [57,58]. This GEM-based Chromium instrument can reach 65% cell capture efficiency (i.e., the proportion of input cells captured for downstream analysis) with a relatively low doublet rate of 0.9% (two or more cells combined). Additionally, up to eight samples are processed in a microfluidic chip capturing 100–80,000 cells in 10–20 min [27,55,56], and the subsequent barcoded libraries are pooled for downstream sequencing. In addition to 10x Chromium, single cells can also be encapsulated with a bead coated with oligonucleotides containing unique molecular identifier (UMI) sequences using the Dolomite-Bio Nadia system. Unlike the GEM-based Chromium system, the Nadia system reverse transcribes RNA into cDNA after the collection of droplets from the chip, providing an advantage in reducing the risk of RT inhibition [59,60]. The Nadia system also provides chips that have integrated stirrers to ensure cells and beads are evenly distributed throughout the run (2–8 samples in a chip per run) [61]. However, the cell capture efficiency is lower when compared to the 10x Chromium system, and the Nadia instrument requires highly trained personnel to operate the run. Wider adoption of these techniques can be limited by the large volume of data that is generated from the sequencing, and therefore the companies are now offering an end-to-end solution for biologists with no prior bioinformatics experience to process, analyse and visualise single-cell expression data.

The Nadia platform does not provide software for data processing and visualisation and requires users to use a publicly available pipelines for analysis. This may be a barrier for widespread use and uptake by biologists, as the publicly available pipeline requires computational skills in R or Unix. For both Chromium and Nadia system, one should consider the requirement for a high concentration of viable cells to maximise the throughput of encapsulation of single cells in droplets. Of all the single-cell instruments, the 10x Chromium system is currently the most popular platform for single-cell analysis in cancer immunotherapy.

### 2.2. Microwell Encapsulation Platforms

The commonly available microwell encapsulation platforms are Fluidigm C1, Illumina/Bio-Rad ddSeq, Takara-Bio ICell8 and BD Rhapsody. Among all the microwell technologies, Fluidigm C1 system was the earliest generation and is considered the founder of the single-cell field. Released in 2012, the C1 system was the first machine that allowed researchers to isolate, select, phenotype and sort single cells for not only whole transcriptome sequencing, but also targeted DNA or RNA sequencing, whole-genome or exome sequencing, small RNA profiling, and epigenomics. The C1 system utilises an integrated microfluidic chip (IFC) to isolate single cells into individual microchannels enabling a cell capture efficiency of 39% [56]. Although the workflow is laborious including manual pipetting and dislodging cells, the C1 instrument allows researchers to visually inspect captured cells under the microscope decreasing doublet rate to 3%. As the IFCs come in the ranges of 5–10, 10–17, and 17–25 µm, the cost for cartridges and reagents can increase substantially especially in the studies of tumour immunology as the cell sizes vary across different cell populations.

In comparison to the C1 system, the Takara-Bio ICell8 platform [62] may be more practical in immuno-oncology studies as ICell8 uses a nano-well chip that captures cells from 5 to 100 µm in size (37% capture efficiency). The ICell8 also provides a customisable workflow where users can visually control for empty wells or doublets and select cells of interest for downstream transcriptomic work. The ICell8 application kit protocol offers the flexibility of different sequencing kits (including Oxford Nanopore Library Preparation Kit and Illumina full-length transcriptome kit) and is compatible with the use of barcoding and UMI for library construction.

Another platform that provides flexibility for single-cell workflow is the Illumina/Bio-Rad ddSeq instrument. The ddSeq scalable kits accommodate both sample size experiments, where one kit is formatted to process hundreds to thousands of cells and the other kit is designed for tens of thousands of cells [63,64]. The protocols for ddSeq are straight-forward, and captured cells are encapsulated into droplets for cDNA synthesis and library preparation for sequencing [65]. Support for end-to-end workflow, including bioinformatics and user-friendly visualisation tools, are provided and are useful to inexperienced users in assisting them to analyse and interpret single-cell data.

More recently, the latest microwell-based instrument, Rhapsody, produced by BD Biosciences claims that its system enables high singlet capture efficiency of up to 80% depending on the cell types and user handling. However, a few studies demonstrated that the overall cell capture rate is 65% especially samples with different cell types and sizes [66,67]. The Rhapsody platform uses UMI-barcoded magnetic beads capturing up to 40,000 single cells on an array of 200,000 microwells, and is a well-based system similar to Microwell-seq [68,69]. Protocols by Rhapsody offer visual inspection of cartridges and microwells to ensure the quality of the samples is adequate for downstream analysis. An additional feature of the workflow is that the remaining beads can be retained for later use, allowing subsample beads to be used for multiple library preparations and thus reducing the sequencing costs. The Rhapsody platform can be incorporated with BD AbSeq to provide absolute quantification of both protein and mRNA expression levels in single cells [70]. The measurement of both protein and mRNA expression is critical to understanding complex regulation of cells because most of the cell surface markers such as CD4 in T cells have thousands of protein molecules per cell but are only driven by a small number of mRNA transcripts [71,72,73]. A common issue in transcriptomic experiments is the dynamic range of mRNA expression levels [72]. Highly expressed genes such as the ribosomal genes will dominate the reads in the sequencing run, while the lowly expressing transcripts including immune genes will be sparse, thus affecting accurate quantification and resulting in unnecessary sequencing costs. The BD Rhapsody workflow provides an option for users to select a specific panel of mRNAs (for instance, BD Rhapsody Immune Response Panel) and allows enrichment of targets to provide higher sensitivity for detecting rare molecules that may be missed with whole-transcriptome profiling [67,70]. A targeted RNA approach is recommended for validation experiments and not for discovery studies.

### 2.3. Fluorescence-Activated Cell Sorting (FACS)

Aside from the modern technologies for single-cell isolation as described earlier, the traditional FACS-based single-cell approach such as SMART-seq2 [74,75] and MARS-seq [76] is a well-established and standardised technique in the laboratory. Both SMART-seq2 and MARS-seq sort individual cells from the target population into 96- or 394-well plates containing lysis buffer, and the plates can be kept for long periods prior to sequencing [77,78]. These techniques are not restricted by the size or morphology of the cells or the total cell numbers, facilitating experiments with very rare cell populations of interest [79]. While the single-cell protocol of MARS-seq is automated, the assays in SMART-seq2 require manual pipetting into individual wells, thereby making it more tedious, and increasing the technical variability [74,75,76]. SMART-seq2 is not suitable for experiments that require thousands of individual cells, unless liquid handling robots are incorporated into the workflow to reduce pipetting issues. Another distinct difference between SMART-seq2 and MARS-seq is the length of cDNA synthesis [75,79]. SMART-seq2 generates full-length cDNAs and produces improved sequencing coverage across the entire transcriptome, whereas MARS-seq employs a 3’ end of single-cell RNA sequencing method where partial cDNAs are tagged with barcodes and UMIs during the reverse transcription step. Compared to 3’ end-counting mRNA, the full-length transcript se-quencing has advantages in detecting lowly expressed genes and isoforms, and allows for allele-specific expression analysis [80,81]. When adopting SMART-seq2 or MARS-seq in the experiments, users should note that the FACS-based approach provides neither visual imaging inspection of cell quality nor the option to select cells for downstream sequencing. As both techniques generally require pre-defined markers, the phenotype of a rare subpopulation requires a wide range of different markers in multiple combinations that can help to better identify subpopulations and strategies for downstream analysis.

**Table 1:** Specifications of common single-cell sequencing technologies.

Platform	Company/Academic	Method of Single-Cell Capture	Capture Efficiency	Doublet Rate	Number of Captured Cells	Cell Size Restrictions	Analytical Tool	Advantages	Relative Limitations	References
Chromium	10x Genomics	Droplet encapsulation	65%	0.90%	100–80,000	Independent of cell size, but generally up to 50 µm	10x analysis suite including Cell Ranger and Loupe Browser; Seurat R package	Easy to operate; cost effective; intensive support for end-to-end solution; flexible options for multiple applications	High concentration of viable cells required; Little control over cell input	[59,78]
DropSeq (Nadia)	Dolomite-bio	Droplet encapsulation	10%	1.80–11.3%	10^3^–10^4^	None for mammalian cells	Open platform	High throughput; low cost	High concentration of viable cells required; low cell capture efficiency; skills required to operate; minimal support for data processing and analysis.	[60,61]
C1	Fluidigm	Microwell encapsulation	39%	3–30%	96 or 800	5–10, 10–17, or 17–25 µm	Fluidigm Singular Analysis Toolset Software	Full-length transcript; customisable workflow (able to exclude empty wells and doublets)	Limited cell capture; low throughput (up to 96 or 800 cells); high cost of cartridges; relatively long preparation time (two runs per day); fresh tissue or cells required	[56,82]
ddSeq	Illumina/Bio-Rad	Microwell encapsulation	3–4%	5.80%	10^3^–10^4^	None for mammalian cells	Illumina BaseSpace or ddSeeker R package	Easy to operate; flexibility of kits for different number of cells; intensive support for end-to-end solution	High concentration of viable cells required; no users modification; single application (RNA-seq)	[56,78]
ICell8	Takara-Bio	Microwell encapsulation	37%	1.3–4%	1800	5–100 μm	CELLSTUDIO software	Easy to operate; full-length transcript; customisable workflow (able to exclude empty wells and doublets)	Specialised bioinformatic tools required; single application (RNA-seq)	[62,78]
Rhapsody	BD Biosciences	Microwell encapsulation	65%	2–10%	100–40,000	5 to 30 μm	BD Rhapsody Analysis Pipelines and SeqGeq Software	Easy to operate; intensive support for end-to-end solution; simultaneously measure protein and mRNA expression; optimise costs based on subsampling and targeted panels	Low sequencing throughput; custom panel of up to 500 targets	[56,66,67,83]
Smart-Seq2	[75,76]	FACS	80%	1%	No limitation	None for mammalian cells	Open platform	No limitations of cell size, shape or homogeneity; simultaneously measure DNA and RNA; high practicality (uses off the shelf reagents); full-length transcript	No options for barcoding and UMI (no multiplexing and gene quantification of samples); laborious worflow due to numerous pipetting steps	[74,75,84]
MARS-Seq	[77]	FACS	92%	2%	No limitation	None for mammalian cells	Open platform	Automated process; suitable for rare cell sorting; No limitations of cell size, shape or homogeneity	Specialised bioinformatic tools required	[76,78]

Abbreviation: FACS—fluorescence-activated cell sorting.

## 3. Spatially Resolved RNA Technologies

Mapping the subcellular position of the RNA molecule in an intact tissue section is an important step to capture the landscape of intra-cellular functions and biological signalling with single-cell techniques. This facilitates deeper understanding of data and tumour biology such as understanding tumour progression and treatment resistance. Several novel high-dimensional spatially resolved RNA technologies include STARmap [85], seqFISH [86], MERFISH [87], FISSEQ [88,89], Slide-seq [90], Nanostring GeoMx [91], 10x Visium/Spatial transcriptomics [33] and High-definition Spatial Transcriptomics (HDST) [92] are summarised in Table 2. There are different methods available for spatially re-solved transcriptomic approaches and the comparison of each method has been reviewed extensively elsewhere [91,93,94]. Therefore, this section focuses on a subset of these in situ capture-based platforms that are becoming increasingly important and widely ac-cessible for biomarker and therapeutic studies in cancer research.

### 3.1. NanoString GeoMx Digital Spatial Profiler (DSP)

The NanoString GeoMx DSP platform allows multiplexed profiling of RNAs and/or proteins at a single spot in 10–600 µm resolution [91]. This instrument uses antibodies labelled with oligonucleotide barcodes and the probes are spatially barcoded with tags for different RNA species to carry out transcriptional profiling from a sample tissue region. GeoMx DSP depends on fluorescent markers to visually guide the selected regions re-vealing morphology (cell sizes and shapes) and/or transcripts of interests. The collected data can be processed, analysed and visualised using GeoMx Data Centre software. GeoMx DSP has gained wider adoption to date due to the robustness of workflow and support for data visualisation and analysis. Another key advantage of this technology is that, unlike the traditional lapser-capture microdissection, GeoMx does not result in de-struction of tissue and hence RNAs from a series of tissue sections can be re-analysed [91,95,96,97]. As GeoMx is designed to analyse spatial expression of a comprehensive panel of RNAs within user-defined regions of interest (ROI) in a tissue section at a single-cell level, the platform requires prior knowledge of targets (up to 18,000 genes) and its sensitivity limits the resolution of ROI at the level of 20–200 cells [93,98]. Although the workflow of selecting ROI is largely automated, analysis of the whole tissue section is not feasible making unbiased regional analysis difficult.

### 3.2. 10x Genomics Visium

The 10x Genomics Visium Spatial Gene Expression system adapted the Spatial Transcriptomics (ST) concept [33] that combines formalin-fixed paraffin-embedded (FFPE) or fresh-frozen tissue imaging with high-throughput sequencing. The company im-proved ST technology by reducing barcode spacing and improving the spatial resolution to 55 µm. Visium consists of a glass pathology slide, embedded with probes with a spatial UMI and a poly(dT) anchor that allows the binding of the poly(A)-tailed mRNA molecules on the solid surface upon permeabilization [45]. The reverse transcription is performed in situ directly on the slide and the subsequent cDNA complexes are extracted for library generation and sequencing. Of note, the Visium assays currently offers 3’ RNA se-quencing for gene identification [65]. Similar to NanoString GeoMx platform, us-er-friendly graphic interface software (Space Ranger) is available for data analysis and visualisation. Visium also allows co-detection of immunofluorescent protein with whole transcriptome spatial analysis. Although Visium provides spatially resolved whole transcriptome data, the current barcoded regions of 55 µm in diameter may include from 1 to 10 cells [33,99]. Similar to the NanoString GeoMx technology, the Visium limits the detection range from a few to hundreds of cells within a given region. Users should note that this may pose some difficulties in profiling certain regions of the TME as cancer cells are frequently adjacent to a combination of immune and stromal cells.

### 3.3. Slide-Seq

Slide-seq, a recently established non-commercial capture-based technique, provides an experimental design similar to that of 10x Genomics Visium, however with a higher cellular resolution of 10 µm [90]. The Slide-seq approach uses a glass coverslip containing uniquely barcoded 10 µm beads that are randomly overlayed. The positions of barcoded beads are decoded in situ by sequencing-by-ligation prior to the sample preparation procedure [94]. Slide-seq technology is useful for profiling large tissue sections as this approach is not bounded by the ROIs [100]. The spatial transcriptomic data generated from Slide-seq requires open-source tools for computational processing and data interpretation, and thus it requires specialised analytics and expertise for data analysis. A key limitation of Slide-seq is that the experimental procedure starting from tissue permeabilization to cDNA extraction for library preparation is relatively time-consuming, which results in the loss of gene expression information due to confounding effects [90]. The reduced sensitivity impacts the ability to detect lower expressed genes, which could impact the biological questions that can be interrogated, particularly those relating to intra- and inter-tumour heterogeneity, rare immune subsets, and their contributions to immunotherapy resistance and tumour relapse.

### 3.4. High-Definition Spatial Transcriptomics (HDST)

HDST has a similar strategy as Slide-seq but instead uses high-definition spatial barcoding beads of 2 µm in size [92]. Each bead contains barcoded mRNA capture primers, and these beads are randomly deposited in an ordered high-density bead array using a split-pool approach. HDST uses smaller beads than Slide-seq and thus has better spatial resolution from 10 µm to 2 µm when compared to Slide-seq [33,92]. Initial protocols before sample preparation in HDST are similar to Slide-seq, where the locations of the beads are decoded by sequential hybridisation. The shortcomings of HDST are similar to Slide-seq and include the requirement for specialised analytics and bioinformatic expertise and low sensitivity of mRNA capture.

**Table 2:** Specifications of in situ capture spatial transcriptomic technologies.

Platform	Company/Academic	Detection Efficiency	Resolution	Number of Captured Cells	Sample Type	Analytical Tool	Advantages	Relative Limitations	References
GeoMx	NanoString	Not reported	10–600 μm	20–200 cells per ROI	Fresh-frozen or FFPE	GeoMx Data Centre Software	Easy to operate (high level of automation); intensive support for end-to-end solution; Ability to profile protein/RNA; single-cell level	Low efficiency of cell capture when using smaller ROIs; Require user-defined ROIs	[91]
Slide-seq	[87]	0.30%	10 μm	~70,000	Fresh-frozen	Open platform or Seurat R package	Relatively high resolution; scalability; spatial resolution for large tissue volumes	Low sensitivity; minimal support for data processing and analysis	[90]
Visium	10x Genomics	>6.9%	55 μm	1–10 cells per ROI	Fresh-frozen or FFPE	10x Space Ranger	Intensive support for end-to-end solution; coverage across a large area of tissue	User-defined regions contain multiple cells	[99]
High-definition spatial transcriptomics	[89]	1.30%	2 μm	~160,000	Fresh-frozen	Open platform	High resolution	Low sensitivity; minimal support for data processing and analysis	[92]

Abbreviations: ROI—region of interest; FFPE—formalin-fixed, paraffin-embedded.

## 4. Dissecting the Tumour Immune Microenvironment Using Single-Cell Approaches

Cancer often begins with errors in the genome that result in the dysregulation of normal cellular behaviour and promotion of a malignant phenotype. Different transcriptional programs and various stages of cell fate contribute to the establishment of intratumoural heterogeneity (ITH), where subpopulation of tumour cells in the same patient appears between different regions of a tumour or gain clonal advantages and evolve overtime to metastasize and emerge immune escape variants [101]. Further complexity is introduced into the evolutionary processes during metastasis and under the selective pressure of systemic therapies. The tumour must establish a TME with a vasculature and stromal framework to support for its growth, whilst recruiting regulatory and immunosuppressive cells through aberrant intercellular and cytokine signalling to evade the immune system. The selective pressure not only dictate the stromal and immune context of the TME, but also elicit selection pressures on the tumour that mitigate the effects of systemic therapies. To fully understand the biology of tumourigenesis, cancer progression, and response to cancer therapies, one requires investigating the dynamics of both tumoural and TME evolution and their interactions. The application of single-cell techniques in immuno-oncology research is demonstrating promising potential for characterising the features of the TME that influence immunotherapy response and resistance in melanoma and other cancer types as summarised in Table 3 and Table 4.

### 4.1. Dissecting Intra-Tumoural Heterogeneity (ITH)

ITH is a major contributor to therapy resistance and cancer progression [114,115,116]. Subclonal variation in oncogenic alterations is hypothesised to be one of the major causes of therapeutic evasion of certain clones and subsequent relapse [117,118,119,120,121]. For instance, the rare therapy-resistant melanoma cells expressing high levels of AXL (receptor tyrosine kinase) are positively selected after treatment with kinase inhibitors contributing to the development of drug resistance [3]. In addition, a minority of highly specialised cells, such as the cancer-like stem cells, is generally in the quiescent cell states that enable a beneficial environment for tumour cells to maintain tumour growth, metastasise, and resist immune and treatment control [122]. The stem-like tumour phenotype is characterised by the high expression of CD133 and CD44, these cells can also evade immune surveillance by upregulating PD-L1 and CD80 through WNT activity, making them resistant to immune-based therapies [51,123,124,125].

The single-cell RNA-seq (scRNA-seq) platform is capable of detecting tumour subclones and determining the transcriptional states and phenotypic differences between individual cells [126,127,128,129]. An example is the scRNA-seq study on uveal melanoma [46], where primary and metastatic tumours were sampled and single cells were processed via the droplet Chromium system. Using trajectory analysis which infers genetic changes during tumour and immune cell evolution, the transcriptional clonal branches were reconstructed to identify the clonal selection of ploidy and transcriptional programs that enabled immune evasion. These approaches hold great promise in understanding immunotherapy resistance under the selective pressures of treatment. The construction of phylogenetic trees of treated patients will allow the identification of different cell lineages that confer resistance to immunotherapy [127,128].

### 4.2. Diversity of the Tumour Immune Microenvironment

Multi-omics heterogeneity is not a feature limited to tumour cells. In fact, being an immunogenic cancer type, various immune cells make up the TME of melanomas, which imposes microenvironmental selection pressures on tumour evolution and mediate responses to some systemic therapies. Unbiased exploration of the TME with genetic sequencing is increasingly utilised to study the cellular interactions and molecular changes in the TME and as well as the tumour.

One of the most common applications of scRNA-seq is to characterise the repertoire and quantity of tumour-infiltrating lymphocytes (TILs). Distinct subpopulations of T cells and the proportion of TILs defined by scRNA-seq analysis of metastatic melanoma have been correlated with immunotherapy response and patient outcomes [17]. Notably, a higher proportion of exhausted T cells with abnormal activation of metabolic pathways, was correlated with unfavourable prognosis, whereas higher proportions of naïve/memory cells and cytotoxic T cells were associated with good prognosis. In a separate study, analysis on transcriptomic profiles of over 16,000 immune cells from 48 melanoma patients receiving immunotherapy identified two distinct CD8+ T cell states. The association of CD8+ T cells with a specific transcription factor, TCF7, was identified as a predictive marker of response [16]. Furthermore, Li et al. identified the formation of dysfunctional T cell compartments in melanoma where early effector CD8+ T cells transition to exhausted states within the TME, and the intensity of the dysfunctional signature was reflective of tumour reactivity to immune response [94]. In contrast, the formation of B cell dominant tertiary lymphoid structures was shown to reverse T cell exhaustion and lead to improved tumour responsiveness to immune checkpoint inhibitors [102,103]. Predictive and prognostic immune effector cell populations were also identified in scRNA-seq analyses of the breast [109], ovarian [111], lung [112,129], and liver cancers [130].

Recent studies have extended the analysis of immune cell heterogeneity to the myeloid-derived populations in the TME. The tumour resident, monocyte-derived dendritic cells was found to be significantly enriched in melanoma patients who respond to anti-PD-1 [104]. Targeting this antigen-presenting cell population with agonist anti-CD40 antibody in mouse models led to the expansion of effector T cells and implementation of anti-tumour immunity [104]. The scRNA-seq analysis of melanoma patients subjected to checkpoint-based immunotherapies also detected upregulation of γδ T cells and TREM2+ macrophages in non-responders [105]. By characterising innate immune cells, we are gaining greater understanding of immunological priming, which guides the anti-tumour T cell response.

The single-cell techniques are also useful for the investigation of cellular interactions. The scRNA-seq of 33 melanoma tumours interrogated malignant cell states that promoted T cell exclusion, and characterised the genomic features of the cold immunological niche associated with poor immunotherapy response [43]. Single-cell sequencing of over 4600 cells from 19 melanoma patients (including malignant, immune, stromal, and epithelial cells) revealed that the TME greatly influenced the gene expression programs of melanoma cells [3]. Analysis of the T cell receptor (TCR) sequences of 2000 T cells from 15 melanoma samples revealed that the expression of co-inhibitory receptors was correlated with T cell activation and was enriched in expanded T cell clones. The study also identified a link between the abundance of cancer-associated fibroblasts (CAFs), a non-malignant stromal cell type, and the expression of tumour gene signatures. Furthermore, a subset of genes expressed by CAFs was found to increase the proportion of CD4+FOXP3+ regulatory T cells (Tregs) creating an immunosuppressive tumour microenvironment that prevent the tumour-reactive immune responses [131,132]. A mechanism of immunotherapy resistance mediated by subtypes of CAFs was also observed in breast cancer, where clusters of CAFs exhibited upregulation of checkpoints in Tregs, in turn increasing the abundance of other potentially suppressive subtypes of CAF within the TME [110,131]. Together, these studies highlight the opportunities for identifying novel immunotherapy targets offered by single-cell sequencing.

## 5. Use of Single-Cell Analysis to Identifying Biomarkers of Response to Immunotherapies and Novel Drug Targets

Studies have begun to explore the systemic effects of immune checkpoint inhibitors using scRNA-seq to gain a more holistic perspective of their mechanisms of action, and to identify biomarkers of treatment response and resistance.

One popular approach is the pairing of single-cell RNA sequencing and TCR sequencing [133]. scRNA-seq of circulating and intra-tumoural T cells allows for the definition of T cell phenotypes, while TCR sequencing reveals the expansion of matching clonotypes in the blood and TME after immunotherapy treatment [134]. This approach has been used to identify expanded T cell clones that correlate with treatment response and resistance. Specifically, T cells that expressed shared TCR sequences can be present as tissue resident T cells (Trm) or as effector memory T cells (Tem) for at least 9 years after a patient has had a lasting response to immunotherapies [133]. This method of investigating matching T cell populations in blood and tumours has provided novel understanding of the cellular mechanisms of response to immunotherapy. However, the expansion of matching clones is suggestive of extravasation of circulating T cells at the tumour site, and hence recognition of tumour neoantigens cannot be directly derived with this method.

By combining patient-specific scRNA-seq analysis of peripheral immune cell phenotypes and clinical data, Griffith et al. presented a method for estimating the degree of anti-tumoural immune attack and predicting clinical response with a dynamic mathematical model [135]. The novel model provided insights into the immune regulation of tumour growth in responders and non-responders to immunotherapy, demonstrating differences in peripheral interferon signalling and cytotoxic T cell differentiation, and hence recognising them as peripheral blood indicators of anti-PD-1 response. The study was able to uncover the evolution of patient-specific responses during treatment by using samples derived from a cohort of clinical trial patients who had the same treatment regimen and timepoints of sample retrieval. The temporal analysis of peripheral blood samples revealed a lower T cell abundance in non-responders to immunotherapy, and the lack of expansion and effector phenotype differentiation after treatment. Additionally, in line with the theory that the host immune system needs time for establishing antigen recognition and developing the adaptive response, the authors observed a delay in cellular response to immunotherapy compared to chemotherapy, which suggested the time required for immunological changes to occur. This highlights the importance of timepoint selection for immunotherapy research and informs future studies and clinical trials.

Blood sampling is a non-invasive source to explore potential biomarkers for immunotherapies. The use of blood bypasses the limitations of bulk tissue biopsy that may account for inter-tumoural heterogeneity, while offering insights into the systemic immune response [136]. Initial reports of melanoma patients treated with ipilimumab showed that improved overall survival and progression-free survival (PFS) were associated with peripheral immune cell counts from routine blood counts at baseline, including low regulatory immune cell frequencies and high lymphocyte frequencies; clinical benefit was also associated with the dynamic changes in blood markers during immune checkpoint treatment, including the reduction in regulatory T cell population and increased lymphocyte counts [137]. Recent single-cell genomic studies using liquid biopsies also revealed associations between the expansion of peripheral T cell subtypes in greater and response to immunotherapy [106,136]; while scRNA-seq of melanoma cells identified immunotherapy resistant to cells harboured mutational programs that translated to mechanisms of T cell exclusion [43], a separate study identifying memory T cell populations persisted for years in patients with durable responses to immunotherapy [134]. These studies show the exciting opportunities to define the properties and evolution of systemic immunity in immunotherapy responders with non-invasive tissue sourcing. Future prospective studies are required to validate the prediction of clinical benefits in larger cohorts.

The spatially resolved transcriptomics method [33] has been used to study and visualise the distribution of mRNAs within tissue sections of melanoma. This emerging field offers the ability to place cell phenotypes of interest within their spatial context, allowing for analysis of intra-cellular communications and TME niches. The visualisation of the transcriptional landscape within stage III lymph node melanoma metastases identified unique gene expression profiles of distinct histological entities, in particular, special expression patterns of lymphoid cells closely located to the tumour margin is potentially reflective of the genetic profile of the melanoma TME [107]. This technology also offered new insights into B cell functional states, suggesting that tertiary lymphoid structures are involved in immunotherapy response [102]. Likewise, the technology can be used to detect the interaction of ligand and receptor pairs in the TME, as the resolution of these analyses increases, the ability to locate individual cells and their ligand-receptor pairing could provide vital information to the rationalisation of novel immunogenic agents [138]. These spatial techniques have not been widely used in research and clinical assessments due to the high cost and throughput considerations. Nevertheless, the results from recent studies as well as the ongoing technological improvements hold promising potential for their future application.

## 6. Future Perspectives in Incorporating Single-Cell Analysis into Clinical Trials and Routine Care of Cancer Patients

In modern cancer treatment, accurate diagnosis and therapeutic decisions are based on the anatomical origin of the primary tumour and its specific features [139,140]. Single-cell analysis by flow cytometry and immunohistochemistry have been utilised for subclassifying haematological malignancies for many years. They are indispensable tools for differentiating non-malignant cells and cancer cells both in the laboratory and in the clinic [21,23]. Single-cell transcriptomic techniques provide a more powerful approach to extensively characterise the entire molecular phenotype of cell populations within biopsies, which could lead to more accurate diagnoses and phenotyping of a patient’s disease. For example, Cohen et al. employed scRNA-seq (MARS-seq) as part of the KYDAR trial (a single-arm prospective trial of daratumumab, carfilzomib, lenalidomide, and dexamethasone (DARA-KRD). The co-authors reported that the transcriptional programs and resistance signatures identified by MARS-seq could assist in the prediction of resistance and guide therapeutic selection based on the specific phenotype of the patients disease [141]. Although single-cell transcriptomic technologies have not been utilised in melanoma clinical trials to date, the insights arising from TME profiles and the tracking of immune cell populations will provide a blueprint for optimising treatment selection and verifying therapeutic effects may improve patients management in the future.

There are still several obstacles that need to be overcome before making these single-cell platforms more readily accessible in the clinic. Firstly, the high dimensionality of single-cell transcriptomic and spatial data requires specialised teams to generate and perform the subsequent bioinformatics analysis. Secondly, sample collection which often requires tumour dissociate especially for scRNA-seq is often limited and logistically challenging. However, spatial transcriptomics on FFPE samples is improving which could mitigate these limitations. Thirdly, the ability to use single-cell transcriptomic platforms to detect low levels of gene expression and transcripts from somatic copy number variants in each cell remains challenging. Lastly, the costs and turnaround times of the current technologies will need to be reduced to operate within clinical expectations.

## 7. Conclusions

Single-cell transcriptomics has uncovered new crucial factors and phenotypic alterations that not only promote tumour progression but also result in therapeutic resistance. In addition, identifying rare subpopulations of cells through single-cell profiling have provided useful insights into response and resistance to treatments. In respect to melanoma and other cancer types, single-cell transcriptomic approaches have paved the way for the discovery of multi-dimensional biomarker signatures associated with immunotherapy response and resistance, and will assist in the development of next-generation immunotherapies which may improve survival outcomes in cancer patients.

## Figures and Tables

**Table 3:** Translational insights of immuno-oncology in melanoma from single-cell analyses.

Key Findings	Single-Cell Platforms	Identified Cell Types	References
CD8 T cells associated with *TCF7* transcription factor were predictive of immunotherapy response; exhausted T cells with abnormal activation of metabolic pathways are correlated with unfavourable prognosis	Smart-Seq2	CD8+ T cell subtypes (exhausted, naïve and cytotoxic)	[16]
Dysfunctional CD8 T cells form a proliferative compartment within human melanoma; the abundance of dysfunctional T cells is associated with tumour recognition	MARS-Seq	Intratumoural CD4 and CD8 T cells	[4]
B cells and tertiary lymphoid structures promote ICB response and improve patient survival	Smart-Seq2	B cells	[102,103]
Monocyte-derived APCs are central to the response of PD-1 checkpoint blockade and anti-CD40 is a potential novel treatment	Smart-Seq2	Monocyte-derived dendritic cells	[104]
Macrophage and γδ T cell subtypes are overrepresented in non-responders to immunotherapy; gene expression signature of these innate cells can help predict treatment response.	Smart-Seq2 and 10x Genomics Chromium	TREM-high macrophages and γδ T cells	[105,106]
A cancer-associated transcriptional program promotes T cell exclusion and resistance to checkpoint immunotherapies	Smart-Seq2	Melanoma cell (resistance signature associated with T cell exclusion and immune evasion)	[43]
Genetic heterogeneity in Stage III melanoma; coexistence of multiple melanoma signatures within a single tumour region	10x Genomics Visium	Gene expression profiles of melanoma and lymphoid cells	[107]
Seven major subpopulations of CD8+ T cells are identified, of which, the exhausted T cell subpopulation is associated with unfavourable prognosis and increased in later-stage melanoma samples, while favourable naïve/memory and cytotoxic subpopulation cells are decreased	10x Genomics Chromium	7 representative subpopulations of CD8+ T cells	[17]

Abbreviations: APCs—antigen-presenting cells; ICB—immune checkpoint blockade; PD-1—programmed cell death 1; γδ—γ delta.

**Table 4:** Translational insights of immuno-oncology of other cancer types from single-cell analyses.

Cancer Type	Key Findings	Single-Cell Platforms	Identified Cell Types	References
Breast	Trajectory analysis on longitudinal samples demonstrated distinct T cell states associated with activation, hypoxia and terminal differentiation	10x Genomics Chromium	CD45+ immune cells (Clusters of T cell, myeloid cell, B cell and NK cell)	[108]
	Tumours with high TILs contained CD8+ T cells with features of TRM T cell differentiation and these CD8+ TRM cells expressed high levels of immune checkpoint molecules and effector proteins; CD8+ TRM gene signature significantly associated with improved patient survival	10x Genomics Chromium	TREM-specific CD8+ T cells	[109]
	Cancer associated fibroblast clusters are linked to immunotherapy resistance, promote cancer cell differentiation and T cell exclusion	10x Genomics Chromium	Cancer-associated fibroblast subsets	[110]
Ovarian	Immune-desert tumours demonstrated low antigen presentation and enrichment of monocytes and immature macrophages; immune-infiltrated and -excluded tumours differ markedly in their T cell composition and fibroblast subsets; chemokine-receptor interactions were identified as potential mechanisms mediating immune cell infiltration	10x Genomics Chromium	Tumour, stromal and immune cells	[111]
Lung	A high ratio of tumour-infiltrating “pre-exhausted” T cells to exhausted T cells was associated with better prognosis; a gene signature of activated tumour Tregs correlated with poor prognosis in lung adenocarcinoma	Smart-Seq2	Peripheral blood, peritumoural and intratumoural T cells	[112]
Liver	Tumour-associated macrophages suppress T cell infiltration in hepatocellular carcinoma and TIGIT-NECTIN2 interaction regulates the immunosuppressive environment; transition of immune cells towards a more immunosuppressive and exhaustive status exemplifies the overall cancer-promoting immune landscape	10x Genomics Chromium	Tumour and immune cells	[113]

Abbreviation: TRM—tissue-resident memory.

## Data Availability

Not applicable.
